# LemonDet: A Lightweight YOLO11 Architecture with Dataset-Specific Color Priors for Small Lemon Detection in Orchards

**DOI:** 10.3390/s26185874

**Published:** 2026-09-16

**Authors:** Sibel Kaplan, Zeki Yetgin, Muhammed Telceken

**Affiliations:** 1 Institute of Science, Department of Computer Engineering, Mersin University, 33343 Mersin, Türkiye; sibelkaplan@mersin.edu.tr; 2 Faculty of Engineering, Department of Computer Engineering, Mersin University, 33343 Mersin, Türkiye; zyetgin@mersin.edu.tr; 3 Faculty of Technology, Department of Computer Engineering, Sakarya University of Applied Sciences, 54187 Sakarya, Türkiye

**Keywords:** small object detection, YOLO11, parameter-efficient object detection, color prior

## Abstract

Accurate and reliable fruit detection in agricultural fields is crucial for applications such as yield estimation, crop tracking, and autonomous harvesting. However, the high density of small fruits in images, overlap, and complex vegetation can limit object detection performance. In this study, LemonDet, a parameter-efficient object detection model based on YOLO11n, is proposed to improve the detection of small lemons in particular. Considering the object scale distribution in the dataset, the standard P3–P5 detection structure is restructured as P2–P4 to ensure the preservation of high-resolution spatial features. In addition, the Lemon Color Prior Convolution (LCP-Conv) module, which transfers the dataset-specific RGB color prior obtained from labeled lemon regions in the training data to the early feature extraction process, has been developed. A label-guided local image enhancement approach, applied only to training images, has also been included in the model to strengthen the limited pixel representations of small objects. In the experimental results, LemonDet achieved 87.2% Precision, 81.6% Recall, 84.3% F1-score, 89.2% mAP50, and 54.8% mAP50-95. With 0.99 million parameters, the model provides a more parameter-efficient architecture than the baseline YOLO models while achieving higher detection performance. Ablation results show that color normalization and label-guided local enhancement contribute to performance. The findings indicate that considering object scale distribution and dataset-specific color information together in model design is an effective approach for detecting small lemons.

## 1. Introduction

In precision agriculture applications, the automatic detection and counting of fruits is important for many applications such as yield estimation, monitoring of crop development, autonomous harvesting, robotic picking, and orchard management [1,2]. The time-consuming and labor-intensive nature of traditional manual counting methods has increased interest in image processing and deep learning-based automatic detection systems [1]. In particular, real-time object detection models, which can provide high detection accuracy with low computational cost, are widely used in smart agriculture and autonomous agriculture systems [2,3,4]. Reliable detection of lemons in orchard environments is a challenging computer vision problem due to complex backgrounds, variable lighting conditions, shading, partial occlusion, and appearance changes depending on different maturity levels [2,3,4,5]. In addition, the representation of lemons as small objects in images with a wide field of view makes it difficult to learn discriminative features due to limited pixel information [5,6]. Sequential downsampling operations performed in convolutional neural networks can reduce the spatial resolution of feature maps, leading to the loss of detailed spatial information, especially for small objects [6,7]. Therefore, preserving high-resolution features and effectively representing object-specific visual information are crucial in the detection of small and partially obscured fruits.

YOLO (You Only Look Once) has been proposed as a single-stage object detection approach that handles object detection as location and class estimation within a single neural network [8]. The YOLO family is widely used in agricultural object detection studies due to the balance it provides between processing speed and detection accuracy [2,3,4]. Current studies show that multiscale feature fusion, attention mechanisms, lightweight network designs, and architectural adjustments for small objects in YOLO-based architectures can improve detection performance in natural environmental conditions [2,3,4,9]. However, in general-purpose object detection architectures, statistical information specific to the target object class or dataset is often not used as an explicit component of the feature extraction process. In addition, the inability to adequately preserve high-resolution features of small objects in the later layers of the network can limit the representation and detection performance of these objects [6,7,9]. Color is one of the important visual features that can be used to differentiate lemons from surrounding plant components in orchard environments. It has been shown that integrating prior information obtained from category-specific color distributions with CNN-based features can support object detection [10]. However, varying lighting and shading conditions can cause significant differences in the color distributions of target objects [11]. Although image enhancement methods can reduce the effect of this variability, it has been reported that the increase in the visual quality of the image does not always directly reflect on object detection performance [12,13]. Therefore, how color information of target objects is used in image preprocessing and feature extraction stages is important for object detection performance. Standard convolution layers learn color information implicitly from training data. In contrast, explicitly including statistical information obtained from target objects in the model allows the learned features to be supported by problem-specific prior knowledge [14]. However, the integrated approach to high-resolution feature representation for small objects, regional image enhancement, and dataset-specific color prior knowledge for lemon detection in natural orchard environments has been limited in the literature.

In this study, a parameter-efficient object detection architecture based on YOLO11n [15], called LemonDet, is proposed for the detection of small lemons in orchard environments. LemonDet combines dataset-specific RGB statistics obtained from target lemon regions, local image enhancement for small object regions, and the use of high-resolution features within the same detection approach. The Lemon Color Prior Convolution (LCP-Conv) module was developed to incorporate color prior information into the early feature extraction process, and detection levels were rearranged to support the representation of small objects.

The main contributions of this study can be summarized as follows:

- A dataset-specific color prior was created based on RGB channel statistics obtained from target lemon regions, and this information was used in image normalization and feature extraction.
- A local image enhancement method based on label information and the LCP-Conv module, which incorporates the color prior into early feature extraction, was developed to enhance visual details in small lemon regions.
- A parameter-efficient LemonDet architecture designed for small-object detection was proposed, combining dataset-specific color information, the LCP-Conv module, and high-resolution feature levels.

## 2. Related Work

Deep learning-based object detection methods are widely used in precision agriculture applications such as fruit detection, counting, and yield estimation. However, variable lighting, obscuration, complex backgrounds, color differences, and small object sizes in orchard environments can limit detection performance [1,16]. The literature directly related to this study was addressed on two main axes: deep learning-based fruit and small object detection and image enhancement, and prior knowledge-based feature learning approaches.

### 2.1. Deep Learning-Based Fruit and Small Object Detection

Deep learning methods offer effective solutions for fruit detection in orchard environments where scale changes, obscuration, and lighting differences are encountered. Koirala et al. [1] comprehensively reviewed deep learning-based fruit detection and yield estimation studies and evaluated the use of CNN-based methods in different fruit types and imaging conditions. Xiao et al. [16] highlighted small objects, obscuration, multiscale feature extraction, and real-time operation requirements as key research problems in fruit detection studies for automated harvesting. YOLO [8] a single-stage object detection approach, is widely used in agricultural applications due to its balance between processing speed and detection accuracy. Mirhaji et al. [17] evaluated YOLO models for orange detection and crop load estimation under different imaging and lighting conditions. In current studies, in addition to accuracy, reducing model complexity and improving real-time applicability are also emphasized. Yang et al. [18] developed a lightweight and efficient fruit detection model in complex garden conditions, while Zhu et al. [19] addressed the ripeness detection of *Camellia oleifera* fruits by developing a lightweight YOLO architecture with attention and multiscale feature processing components. These studies show that architectural improvements in agricultural object detection mostly focus on lightweight network design, attention mechanisms, and multiscale feature processing. Since a significant portion of fruits in garden images can occupy small areas in the image, small object detection is of particular importance for these applications. The limited pixel area of small objects makes it difficult to represent texture, edge, and shape information adequately; low resolution, occlusion, and background complexity also exacerbate this problem [5,20]. Feng et al. [20] and Nikouei et al. [5], in their studies on small object detection, considered multiscale feature extraction, high-resolution feature preservation, attention mechanisms, and feature fusion as the main solution approaches. Sequential downsampling operations in convolutional networks can reduce the spatial resolution of feature maps, leading to the loss of detailed information about small objects. The Feature Pyramid Network (FPN) proposed by Lin et al. [7] combines high-resolution spatial information with deeper semantic representations by linking features at different network levels through top-down and lateral connections. This approach has also been a key component of later small object-oriented YOLO studies. Feng et al. [21] aimed to more effectively utilize high-resolution feature levels by redesigning the YOLOv8 architecture for small object detection. Wang et al. [22] focused on preserving high-resolution features and improving multiscale feature interaction in the CPDD-YOLOv8 model.

When these studies are considered together, preserving high-resolution early features and effectively combining features at different scales emerge as fundamental design requirements for detecting small objects in natural environments. However, these architectural approaches mostly rely on visual features learned from the image; the explicit inclusion of statistical information specific to the target object or dataset into the feature extraction process is addressed to a more limited extent.

### 2.2. Image Enhancement and Prior Knowledge-Based Feature Learning

In images obtained in natural environments, low contrast, variable lighting, shadowing, and color distortions can negatively affect the distinctive visual features of target objects. Guo et al. [11] investigated low-light image enhancement methods and showed that histogram-based, Retinex-based, and learning-based approaches can be used in different image distortions. Wang et al. [23] evaluated the effect of contrast enhancement-based preprocessing on classification, object detection, and instance segmentation and reported that CLAHE applied with appropriate parameters can be useful in low-contrast regions. Similarly, Shi et al. [24] used CLAHE-based contrast enhancement and color correction together, while Jin et al. [25] evaluated local contrast enhancement and color correction methods together. However, the increase in the perceptual quality of the image does not always directly lead to an improvement in object detection performance. Liu et al. [12] addressed image enhancement and object detection within an integrated structure, enabling the enhancement information to be used by the detection network. Kou et al. [13] evaluated a lightweight image enhancement framework for low-light scenes in conjunction with a YOLO-based detector. These studies demonstrate that image enhancement processes should be evaluated not only in terms of visual quality but also in terms of their effects on the subsequent detection task.

More recently, deep learning-based super-resolution methods have provided advanced approaches for recovering fine spatial details from low-resolution imagery. Xiao et al. [26] proposed TTST (Top-k Token Selective Transformer), a Transformer-based method for remote sensing image super-resolution that dynamically selects informative tokens and combines multi-scale feature aggregation with global context modeling. Xiao et al. [27] also proposed EDiffSR, an efficient diffusion probabilistic model for remote sensing image super-resolution that employs an Efficient Activation Network (EANet) for noise prediction and a Conditional Prior Enhancement Module (CPEM) to extract informative conditional features from low-resolution images. These studies highlight recent advances in Transformer- and diffusion-based super-resolution for recovering fine spatial information from low-resolution imagery.

In the studies reviewed, image enhancement was mostly addressed at the image level, while selective enhancement towards target object regions was examined more limitedly. In addition to image enhancement approaches, the transfer of problem-specific prior knowledge to deep learning models is also used to support the feature learning process. Prior knowledge can be represented in the form of statistical distributions, expert knowledge, physical properties, or object-specific visual features. Mumuni and Mumuni [28] comprehensively evaluated the effects of prior knowledge on representation learning, generalization, explainability, and robustness in deep learning systems. One of the directly related studies on the use of color prior to object detection was carried out by Gu et al. [10]. In this study, adaptive color prior features were created using category-specific color distributions obtained from labeled object regions and scene color information, and this information was integrated with multiscale CNN features. This approach has shown that color information can be used as an additional prior in object detection. However, in this method, the color prior is represented in the form of prominence-based feature maps. Regarding the use of prior information in YOLO-based architectures, Li et al. [29] combined prior information fusion with an improved YOLOv5 architecture in tiger fish detection. The study aimed to strengthen the representation of fuzzy and densely overlapping targets and applied a prediction box aggregation mechanism in addition to prior information fusion. In the Prior Sparse R-CNN approach proposed by Qian et al. [30], the global object information obtained by the Region Generation Network is integrated with regional feature representations. Thus, it has been shown that prior information can be included in object detection models in different forms, such as color distribution [10], target appearance [29], and global semantic features [30].

These studies demonstrate that prior knowledge is an additional information source that can support the standard data-based feature learning process. However, the source of the prior knowledge used, its representation format, and the integration point within the network differ among the methods. When the literature is evaluated generally, the use of high-resolution features for small objects [5,7,20,21,22], the effect of image enhancement on object detection [11,12,13,23,24,25], and the integration of different types of prior knowledge into detection networks [10,28,29,30] stand out as separate research directions. In contrast, the evaluation of dataset-specific color information obtained from target object regions, together with regional image enhancement and high-resolution feature representation, within the scope of small lemon detection has remained limited.

## 3. Materials and Methods

**Figure 1:**
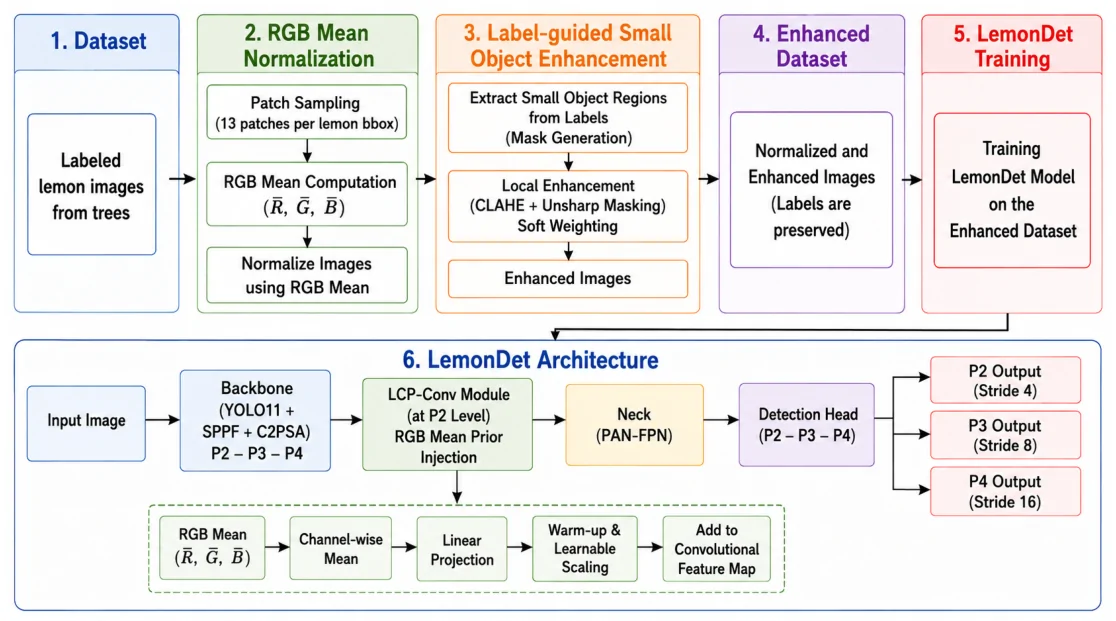
General workflow and architectural structure.

The proposed LemonDet approach is based on integrating high-resolution feature representation for small objects with the use of dataset-specific color information in image preprocessing and feature extraction processes. The overall workflow and basic components of the method are shown in Figure 1.

The proposed approach consists of two interrelated core components: data preprocessing and the LemonDet object detection architecture. In the data preprocessing phase, RGB channel averages specific to the dataset are calculated using image segments sampled from labeled lemon regions. The resulting color statistics are used in RGB-based normalization to reduce color distribution differences between images. Then, regions corresponding to small lemon objects are identified using label information, and selective image enhancement operations are applied to these regions to strengthen local contrast and edge information. The resulting images, while preserving the original object labels, are used in training the LemonDet model.

### 3.1. Dataset

**Figure 2:**
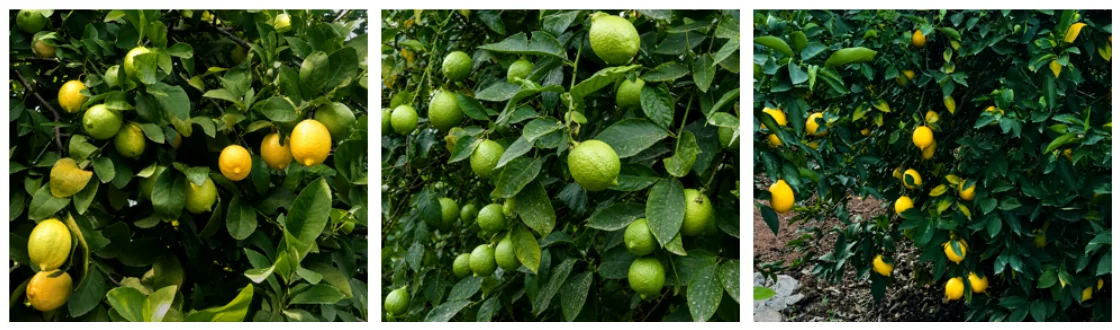
Sample lemon images selected from the dataset, including different perspectives, object scales, lighting conditions, backgrounds, and appearance differences.

In this study, a unique dataset was created to represent different viewing perspectives, object scales, lighting conditions, and variations in the appearance of lemons. Images were obtained from different lemon orchards in Mersin province using Unmanned Aerial Vehicles (UAVs) flown at both ground level and different altitudes. Image frames were extracted from the videos recorded during the UAV shoots and included in the dataset. The combined use of ground-level and aerial shots allowed for the representation of lemons at different camera-object distances, viewing angles, scales, backgrounds, and viewing conditions. Sample images selected from the dataset are presented in Figure 2.

The dataset consists of 9469 labeled lemon objects within a total of 436 images. While there are approximately 21.7 lemon objects per image on average, the number and scale of objects in the image vary depending on camera-object distance, viewing perspective, and shooting conditions. To increase the visual diversity of the dataset, lemon samples with different maturity levels and associated color and appearance differences have also been included.

Special attention was paid to maintaining independence between the training and validation subsets. Rather than randomly distributing all images, the subsets were constructed using images obtained from different acquisition sequences. Images from the same or visually highly similar shooting sequences were not included in both subsets. In particular, consecutive or highly similar frames extracted from the same UAV video were assigned to only one subset. The validation subset also included different shots, orchard views, and imaging conditions from those represented in the training data. This sequence-level separation was used to reduce visual and temporal overlap between the training and validation data.

As a result of this separation, the training set consists of 8524 labeled lemon objects within 375 images, while the validation set consists of 945 lemon objects within 61 images. Accordingly, approximately 90% of the labeled lemon objects are in the training set, and 10% are in the validation set. The distribution of the dataset according to the training and validation sets is given in Table 1.

Before model training, all images were resized to 640×640 pixels to match the network input size. Data augmentation was applied only to the training set, with three augmented outputs generated per training example. The augmentation procedure included horizontal flipping, $90^{\circ }$ clockwise and counterclockwise rotations, random noise affecting up to 0.34% of the image pixels, and Mosaic augmentation. These operations were used to increase visual variability in the training data and reduce the model’s dependence on specific object orientations and acquisition conditions. Following augmentation, the effective training data contained 25,626 annotated lemon instances. No data augmentation was applied to the validation set, which was kept unchanged for performance evaluation.

### 3.2. Obtaining the Dataset-Specific RGB Color Prior

To obtain the dataset-specific RGB color prior representing the channel characteristics of lemon objects, a patch-based statistical analysis was performed using annotated lemon regions from the training data. Both the original training images and the augmented training samples were included in the computation of the color prior, allowing the resulting statistics to represent the color variability present in the data used for model training. Images and annotations from the validation set were excluded from this computation. Thus, the color prior was derived exclusively from the training data, preserving the independence of the validation set.

**Figure 3:**
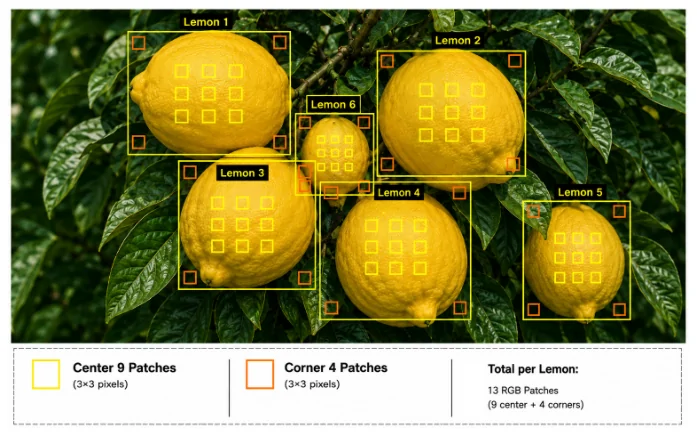
Patch sampling strategy applied to an annotated lemon region. A total of 13 RGB patches of size 3×3 pixels are sampled within each bounding box, comprising nine central patches and four patches positioned near the corner regions.

A total of 13 RGB patches of size 3×3 pixels were sampled from each annotated lemon region to capture color information from different spatial locations within the bounding box. Nine patches were sampled from the central region, while four additional patches were sampled near the corner regions of the bounding box. The central patches primarily represent color information from the interior region of the annotated lemon, whereas the corner patches provide additional samples from locations closer to the bounding-box boundaries. The four corner patches were intentionally retained to capture boundary-related color variability associated with illumination, shading, neighboring fruits, and transitions toward surrounding vegetation. All sampled patches were constrained to remain within their corresponding bounding boxes. The spatial arrangement of the sampled patches is illustrated in Figure 3.

Each 3×3 RGB patch contains nine spatial positions for each of the three color channels and is represented by a 27-dimensional vector. The vector corresponding to the *k*th patch is defined in Equation (1):

(1) $$x_{k}={\left[R_{k,1},R_{k,2},\ldots ,R_{k,9},G_{k,1},G_{k,2},\ldots ,G_{k,9},B_{k,1},B_{k,2},\ldots ,B_{k,9}\right]}^{T}\in R^{27},$$

where $R_{k,j}$, $G_{k,j}$, and $B_{k,j}$ denote the red, green, and blue channel values, respectively, at the *j*th spatial position of the *k*th patch, with j∈{1,…,9}.

The patch vectors extracted from all annotated lemon regions in the original and augmented training images were combined into a single feature matrix. Let *N* denote the total number of sampled patches used to compute the dataset-specific RGB color prior. The resulting feature matrix is defined in Equation (2):

(2) $$X=\left[\begin{array}{c} x_{1}^{T}\\ x_{2}^{T}\\ \vdots \\ x_{N}^{T} \end{array}\right]\in R^{N\times 27}.$$

Each row of X corresponds to a 3×3 RGB patch, while its columns represent the channel-specific pixel positions within the patch. The dataset-level mean RGB feature vector is then obtained by averaging all patch vectors according to Equation (3):

(3) $$\mu =\frac{1}{N}\sum_{k=1}^{N}x_{k},\;\mu \in R^{27}.$$

The resulting vector μ represents the mean RGB characteristics of the annotated lemon regions across the original and augmented training samples. To obtain channel-specific statistics, the 27-dimensional mean vector is decomposed into three nine-dimensional subvectors corresponding to the red, green, and blue channels, as defined in Equation (4):

(4) $$\mu =\left[\mu_{R},\mu_{G},\mu_{B}\right],\;\mu_{R},\mu_{G},\mu_{B}\in R^{9}.$$

The nine spatial components associated with each channel are subsequently averaged to obtain the dataset-specific channel means. The mean values for the red, green, and blue channels are calculated according to Equation (5):

(5) $$\bar{R}=\frac{1}{9}\sum_{j=1}^{9}\mu_{R,j},\;\bar{G}=\frac{1}{9}\sum_{j=1}^{9}\mu_{G,j},\;\bar{B}=\frac{1}{9}\sum_{j=1}^{9}\mu_{B,j}.$$

Based on these channel statistics, the dataset-specific RGB color prior is defined in Equation (6) as

(6) $$p_{RGB}={\left[\bar{R},\bar{G},\bar{B}\right]}^{T}.$$

The channel values obtained from the patch-based analysis and constituting the dataset-specific RGB color prior are summarized in Table 2.

The resulting $p_{RGB}$ vector is utilized at two stages of the proposed approach. First, it is supplied to the LCP-Conv module as dataset-specific prior information, enabling the color characteristics derived from the annotated lemon regions to be incorporated into the feature extraction process. Second, the same channel statistics serve as reference values for RGB-based color normalization prior to the local image enhancement stage.

For color normalization, the mean value of each RGB channel is calculated independently for each training image. Rather than directly matching these image-specific channel means to the corresponding dataset-specific reference values, a soft color matching strategy is employed. Let $\mu_{c}$ denote the mean value of channel *c* in an input image and $\mu_{c}^{\mathrm{ref}}$ denote the corresponding reference mean derived from $p_{RGB}$. The target channel mean $\mu_{c}^{\prime }$ is calculated according to Equation (7):

(7) $$\mu_{c}^{\prime }=\mu_{c}+\lambda_{\mathrm{norm}}(\mu_{c}^{\mathrm{ref}}-\mu_{c}),\;c\in \left\{R,G,B\right\},$$

where $\mu_{c}^{\prime }$ denotes the target mean of channel *c* after color normalization and $\lambda_{\mathrm{norm}}$ controls the strength of the adjustment toward the corresponding dataset-specific reference value. In this study, $\lambda_{\mathrm{norm}}$ was set to 0.35. For each channel, 35% of the difference between the image-specific mean and the corresponding reference mean was applied as an additive adjustment to the pixel values of that channel. This value was empirically selected during method development to provide a moderate adjustment toward the dataset-level RGB reference while preserving the original color characteristics of the images and avoiding excessive color alteration. The resulting pixel values were then constrained to the valid intensity range. In this way, the RGB channels were shifted toward the dataset-specific reference statistics without directly replacing the original color information. The normalized training images were subsequently used as input to the annotation-guided local enhancement procedure described in the following section.

RGB-based color normalization was applied only to the training images. It was not applied to the validation images or to unseen images during inference. Thus, validation and inference images were provided to the detector without RGB-based color normalization.

### 3.3. Local Image Enhancement Based on Small Object Annotations

The limited pixel area of small lemon objects in the image can make it difficult to adequately represent the contrast, edge, and texture information of the object in the feature extraction process. To reduce this effect, a local image enhancement approach has been applied that concentrates the effect of visual enhancement in small object regions using object positions obtained from annotations. The proposed process consists of the following steps: determining small objects according to their bounding box areas, creating binary masks and soft weight maps from these regions, strengthening contrast and edge information, and merging the enhanced image with the original image using the weight map.

First, the normalized bounding box coordinates in the YOLO format annotations of the training images were converted to pixel coordinates according to the image dimensions. The area of the object region is calculated using the width $w_{i}$ and height $h_{i}$ of the bounding box obtained for each object in pixels as follows:

(8) $$A_{i}=w_{i}h_{i}$$

Here, $A_{i}$ represents the bounding box area of the *i*th object. The area threshold was set to $T_{\mathrm{small}}=1024\;{\mathrm{pixel}}^{2}$. Accordingly, the set of bounding boxes satisfying the small-object criterion is defined as:

(9) $$B_{\mathrm{small}}=\left\{B_{i}|A_{i}\leq T_{\mathrm{small}}\right\},$$

where $B_{\mathrm{small}}$ denotes the set of bounding boxes whose areas do not exceed the predefined threshold $T_{\mathrm{small}}$. The selected threshold of $1024\;{\mathrm{pixel}}^{2}$ corresponds to an object area of 32×32 pixels and is consistent with the small-object area criterion used in the COCO evaluation protocol [31]. Accordingly, lemon instances with bounding box areas not exceeding $1024\;{\mathrm{pixel}}^{2}$ in the resized 640×640 images were included in $B_{\mathrm{small}}$ and subjected to the proposed local enhancement procedure. Based on this criterion, 17,823 of the 25,626 annotated lemon instances in the augmented training data were classified as small objects, corresponding to 69.55% of the instances used during training. The remaining 7803 instances (30.45%) exceeded the defined small-object threshold. Thus, small objects constituted the majority of the object instances used during model training.

An additional comparison of the object-size distributions before and after data augmentation showed that the overall distribution was largely preserved. Although some augmentation operations, such as Mosaic, can alter the apparent scale of individual objects through image composition and resizing, no substantial change was observed in the overall small-object distribution after augmentation. The difference between the small-object proportions in the training and validation subsets therefore reflects the different object-scale distributions represented by these two subsets. Since the training and validation data were separated at the acquisition-sequence level, variations in camera-to-object distance, viewpoint, and imaging conditions across different acquisition sequences can result in different apparent object-size distributions between the two subsets.

To represent the spatial positions of the defined small object regions on the image, a binary mask with the same width and height as the input image is created. The pixel positions covered by the bounding boxes in the $B_{\mathrm{small}}$ set are assigned the value 1, while the other pixel positions are assigned the value 0. Accordingly, the binary mask is defined as follows:

(10) $$M_{\mathrm{bin}}\left(x,y\right)=\left\{\begin{array}{cc} 1, & \left(x,y\right)\in \underset{B_{i}\in B_{\mathrm{small}}}{⋃}B_{i},\\ 0, & \mathrm{otherwise} \end{array}\right.$$

Since directly using the binary mask could cause abrupt transitions at the boundaries of the selected object regions, Gaussian smoothing was applied to $M_{\mathrm{bin}}$. This results in a continuous weight map with high values at the center of small object regions and gradually decreasing values as one moves away from the object boundaries. This process is expressed as:

(11) $$M_{\mathrm{soft}}=G_{\sigma }*M_{\mathrm{bin}}$$

**Figure 4:**
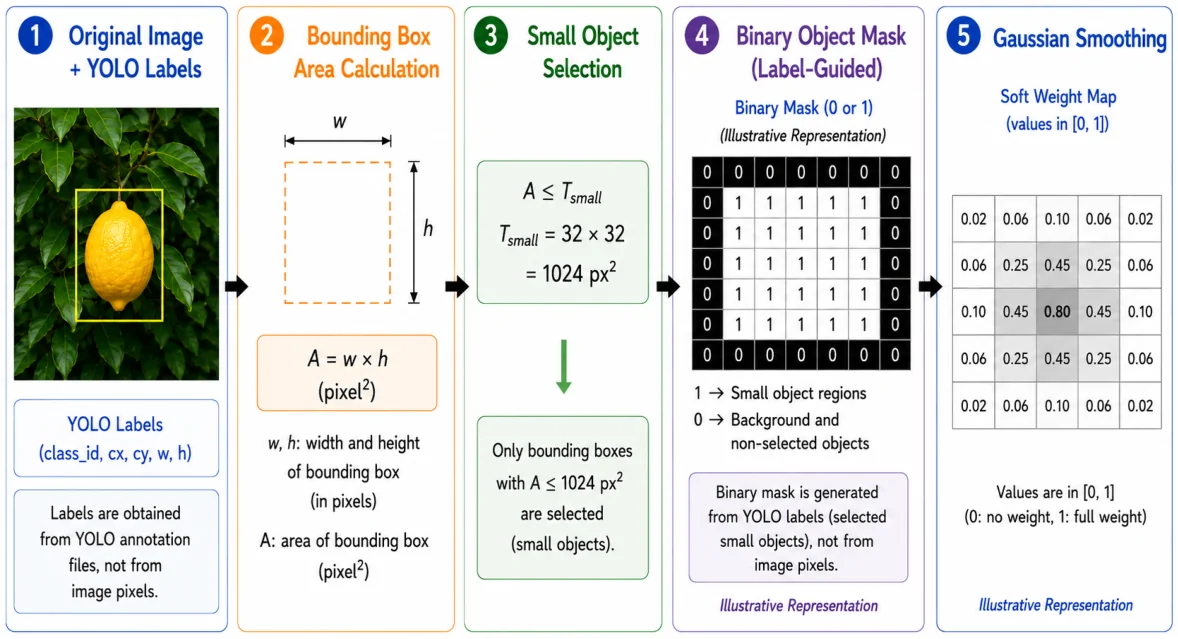
Annotation-based small-object selection and soft weight map generation. Bounding box areas are evaluated using the threshold $T_{\mathrm{small}}=1024\;{\mathrm{pixel}}^{2}$, a binary mask is generated from the selected small-object regions, and the continuous soft weight map $M_{\mathrm{soft}}$ is obtained by Gaussian smoothing.

Here, $G_{\sigma }$ denotes the Gaussian kernel with standard deviation σ, ∗ denotes the convolution operation, and $M_{\mathrm{soft}}$ denotes the resulting continuous soft weight map. In the implementation, a 31×31 Gaussian kernel was used for mask smoothing, while the Gaussian standard deviation σ was automatically determined by OpenCV from the specified kernel size. The smoothing operation gradually reduces the enhancement weights toward the boundaries of the selected regions, thereby avoiding abrupt spatial transitions during the subsequent blending stage. Thus, the effect of the image enhancement performed in the next stage is concentrated in small object regions and smoothly reduced towards the periphery of these regions. The workflow for identifying small objects from annotations, creating the binary mask, and obtaining the $M_{\mathrm{soft}}$ weight map as a result of Gaussian smoothing is shown in Figure 4.

To enhance the visual details of small objects, contrast enhancement and edge sharpening were applied together. To limit the effect of contrast adjustment on color components, the RGB image was converted to the CIELAB color space, and Contrast Limited Adaptive Histogram Equalization (CLAHE) was applied only to the *L* channel, which represents the brightness information. The *a* and *b* color components were preserved unchanged. The brightness channel obtained after CLAHE is defined as:

(12) $$L_{\mathrm{enh}}=\mathrm{CLAHE}(L)$$

Here, *L* represents the brightness channel of the input image, and $L_{\mathrm{enh}}$ represents the brightness channel to which CLAHE was applied. In the implementation, CLAHE was applied with a clip limit of 1.5 and a tile-grid size of 8×8. The resulting $L_{\mathrm{enh}}$ channel was recombined with the unchanged *a* and *b* channels, and the image was converted to the RGB color space.

Unsharp masking was applied to enhance object boundaries and local texture details after contrast enhancement. The resulting image from the CLAHE process is $I_{\mathrm{clahe}}$, and its Gaussian-blurred version is $I_{\mathrm{blur}}$. The enhanced image is calculated as follows:

(13) $$I_{\mathrm{enh}}=1.10\;I_{\mathrm{clahe}}-0.10\;I_{\mathrm{blur}}$$

In the implementation, the Gaussian blur used for unsharp masking was applied with σ=1.0, while the original and blurred components were weighted by 1.10 and −0.10, respectively. These settings were kept fixed throughout the experiments.

The resulting $I_{\mathrm{enh}}$ was not used directly as the output image. Instead, the previously created weight map $M_{\mathrm{soft}}$ was used to concentrate the spatial effect of contrast and sharpness enhancement on small object regions.

In the final stage, the original image $I_{\mathrm{orig}}$ and the enhanced image $I_{\mathrm{enh}}$ are combined at the pixel level using the weight map $M_{\mathrm{soft}}$. The merging process is defined as follows:

(14) $$I_{\mathrm{out}}\left(x,y\right)=\left[1-\lambda_{\mathrm{blend}}M_{\mathrm{soft}}\left(x,y\right)\right]I_{\mathrm{orig}}\left(x,y\right)+\lambda_{\mathrm{blend}}M_{\mathrm{soft}}\left(x,y\right)I_{\mathrm{enh}}\left(x,y\right)$$

**Figure 5:**
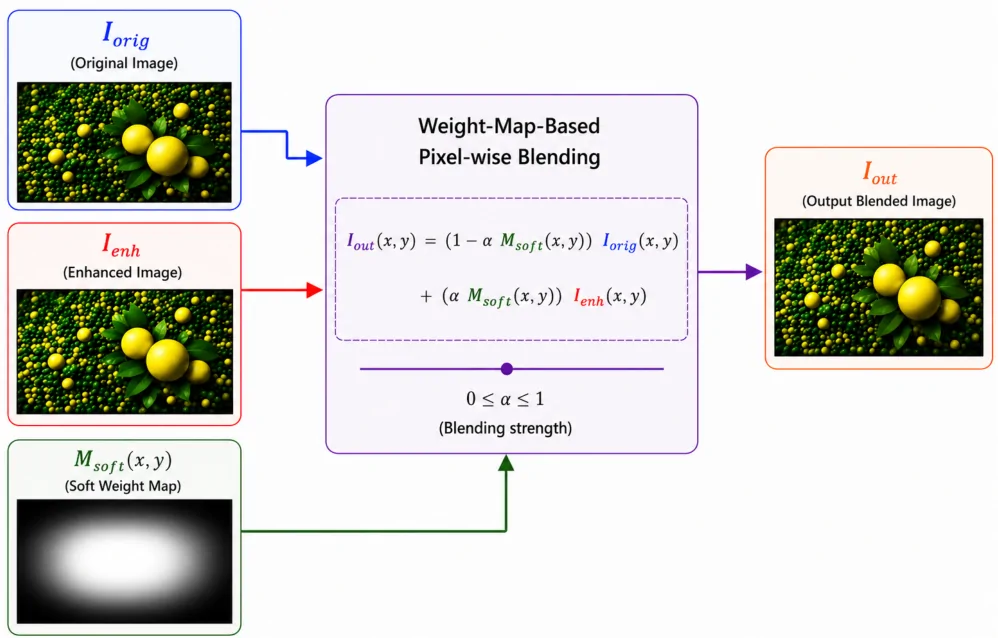
Pixel-level blending of the original image $I_{\mathrm{orig}}$ and the enhanced image $I_{\mathrm{enh}}$ using the soft weight map $M_{\mathrm{soft}}$ and blending coefficient $\lambda_{\mathrm{blend}}$.

Here, $I_{\mathrm{out}}\left(x,y\right)$ represents the output image, $M_{\mathrm{soft}}\left(x,y\right)$ represents the local enhancement weight at the corresponding pixel location, and $\lambda_{\mathrm{blend}}$ represents the blending coefficient controlling the contribution of the enhanced image. In this study, $\lambda_{\mathrm{blend}}=0.8$ was used. This value was empirically selected during method development to provide sufficient enhancement within small-object regions while maintaining smooth transitions between the enhanced regions and the surrounding unprocessed areas. When $M_{\mathrm{soft}}\left(x,y\right)$ approaches zero, the output pixel is predominantly determined by $I_{\mathrm{orig}}\left(x,y\right)$, while as the weight value increases, the contribution of $I_{\mathrm{enh}}\left(x,y\right)$ to the output gradually increases. Thus, the effect of contrast and edge enhancement is increased in small-object regions, while the original image is largely preserved outside these regions. The use of a soft weight map also prevents the formation of sharp boundaries between enhanced and preserved regions, providing a more continuous transition. The applied pixel-level blending process is shown in Figure 5.

As a result of this procedure, the geometric structure of the images and their bounding box annotations remained unchanged, while contrast, edge, and local texture information was selectively enhanced in regions corresponding to small lemon objects. The proposed local enhancement procedure was applied exclusively to the training images, and bounding box annotations were used only to identify the regions to be enhanced during training. No annotation-based local enhancement was applied to the validation images, and validation annotations were not involved in any preprocessing operation. The resulting training images were subsequently used to train the proposed LemonDet architecture, while model performance was evaluated on validation images without using prior information about the ground-truth object locations.

## 4. Proposed LemonDet Architecture

In this study, a YOLO11n-based LemonDet architecture was developed to utilize high-resolution features and dataset-specific color information in the detection of small lemon objects. YOLO was proposed by Redmon et al. as a single-stage object detection approach that performs object positioning and classification operations within a single neural network. Subsequent versions of the YOLO family improved the backbone, multiscale feature fusion, and detection structures; in the YOLO11n architecture, feature extraction and multiscale representation structures were redesigned using components such as C3k2, SPPF, and C2PSA.

**Figure 6:**
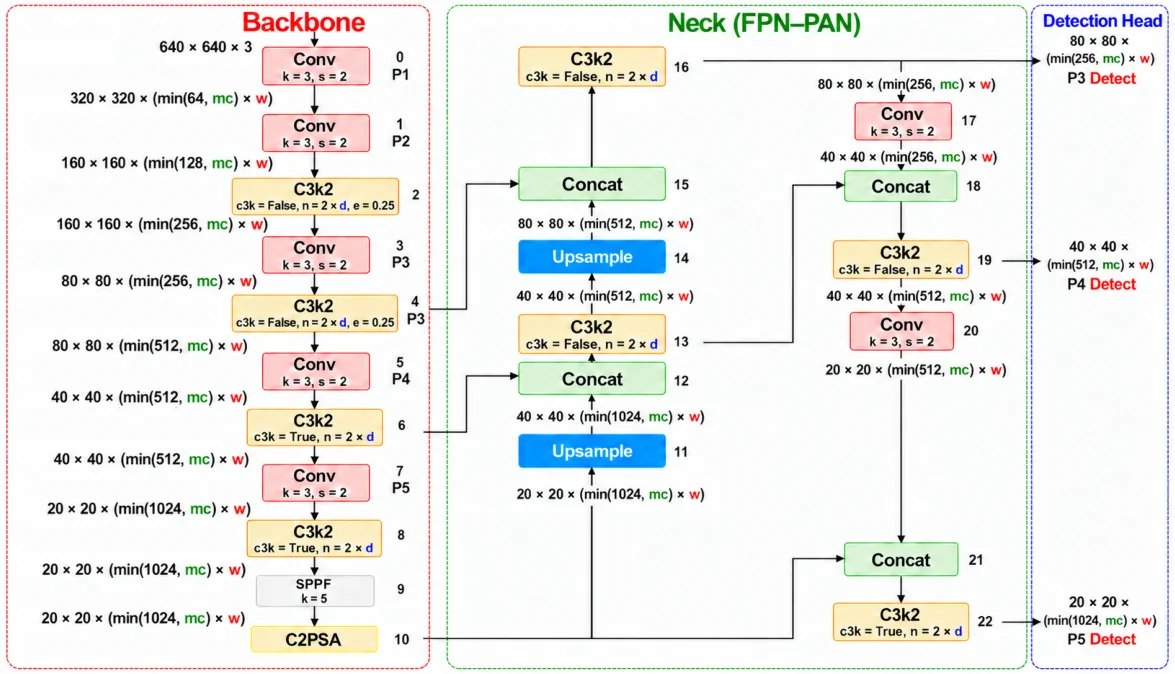
The YOLO11 architecture was used as the basic model in this study.

The YOLO11n architecture, used as the basic model in this study, consists of a backbone that extracts features at different scales from the input image, a neck that combines these features, and a detection head that estimates object classes and bounding box positions [15]. The backbone utilizes C3k2-based feature extraction blocks along with SPPF and C2PSA components. The neck structure leverages Feature Pyramid Network (FPN) and Path Aggregation Network (PAN) principles to combine features at different resolution levels [7,32]. FPN enables the combination of high-level semantic features with high-resolution features, while the PAN structure strengthens the bottom-up feature transfer path, supporting information flow between different feature levels [32]. The standard YOLO11n object detection architecture generates predictions from P3, P4, and P5 feature levels. The basic YOLO11n structure used in this study is shown in Figure 6.

In the LemonDet design, the core YOLO11n architecture has been redesigned in two key areas. First, the Lemon Color Prior Convolution (LCP-Conv) module has been developed and integrated into the high-spatial-resolution P2 level of the backbone to transfer the dataset-specific RGB color prior obtained from the lemon regions in the training data to the feature extraction process. Second, the standard P3–P5 detection levels have been reorganized as P2–P4, allowing the P2 feature map to be used directly in object detection and eliminating the low-spatial-resolution P5 detection level. These two arrangements combine the inclusion of dataset-specific color information in early feature extraction and the transfer of high-resolution features of small objects to the detection stage within the same architecture.

All experiments were conducted using the nano-scale YOLO11n model (YOLO11n) as the baseline architecture. The models were implemented and trained using Ultralytics 8.3.145 with PyTorch 2.11.0 and CUDA 12.8 in a Google Colab environment equipped with an NVIDIA Tesla T4 GPU. The input resolution was fixed at 640×640 pixels, and all models were trained for 100 epochs with a batch size of 8. The optimizer was configured using the automatic selection mode provided by the Ultralytics framework, which selected AdamW with an initial learning rate of 0.002 and a momentum of 0.9. A weight decay of 0.0005 was used, and early stopping was configured with a patience of 20 epochs. A random seed of 0 was used for all YOLO-based experiments to support reproducibility. The same training settings were used for all YOLO-based configurations to maintain consistency across the corresponding comparative experiments.

### 4.1. Lemon Color Prior Convolution (LCP-Conv)

Standard convolution layers transform visual information in the input data into feature maps through filters learned during training. In LemonDet, while this standard feature extraction path is preserved, the aim is to transfer pre-calculated dataset-specific color statistics from the lemon regions in the training data as additional prior information to the feature extraction process. Lemon Color Prior Convolution (LCP-Conv), developed for this purpose, combines the standard convolutional feature extraction path with a parallel prior information path that processes RGB color priors.

The basic feature extraction path of LCP-Conv consists of convolution, batch normalization, and SiLU activation. The standard convolutional feature map generated by this path, where the input feature map is $X\in R^{B\times C_{\mathrm{in}}\times H\times W}$, is defined by Equation (15).

(15) $$Y=\mathrm{SiLU}(\mathrm{BN}(\mathrm{Conv}(X))),\;Y\in R^{B\times C_{\mathrm{out}}\times H^{\prime }\times W^{\prime }}$$

In the equation, *B* represents the batch size, $C_{\mathrm{in}}$ and $C_{\mathrm{out}}$ represent the number of input and output channels respectively, *H* and *W* represent the spatial dimensions of the input feature map, and $H^{\prime }$ and $W^{\prime }$ represent the spatial dimensions of the feature map obtained as a result of the convolution operation. This shows that Y represents the feature representation learned by the standard convolutional path within LCP-Conv.

Parallel to the standard feature extraction path, the second path of LCP-Conv processes the dataset-specific RGB color prior $p_{RGB}$, which is derived from the annotated lemon regions in the training data and defined in Equation (6).

This three-dimensional prior is used as the input to the prior-information branch of LCP-Conv. To integrate it with the $C_{\mathrm{out}}$-channel feature representation generated by the standard convolutional path, a learnable linear projection is applied. This transformation is expressed by Equation (16):

(16) $$p=W_{p}p_{RGB},\;p\in R^{C_{\mathrm{out}}},$$

where $W_{p}\in R^{C_{\mathrm{out}}\times 3}$ denotes the learnable projection matrix. The resulting vector p represents the dataset-specific RGB color prior projected into the $C_{\mathrm{out}}$-dimensional feature space. The projection weights are initialized using the standard initialization of the linear projection layer and are not initialized to zero. The projected vector p is subsequently broadcast across the spatial dimensions $H^{\prime }\times W^{\prime }$ to obtain $\tilde{p}$, making it compatible with the spatial dimensions of the convolutional feature map Y.

To gradually introduce the contribution of the RGB color prior during training, a linear warm-up mechanism is used in LCP-Conv. Let *t* denote the current training iteration and $T_{w}$ denote the warm-up duration. The warm-up coefficient $\alpha_{t}$ is defined as:

(17) $$\alpha_{t}=\min\left(\frac{t}{T_{w}},1\right),\;T_{w}=2000.$$

Accordingly, $\alpha_{t}$ increases linearly from 0 to 1 during the first 2000 training iterations and remains equal to 1 thereafter. During inference, $\alpha_{t}$ is fixed to 1. In addition to the warm-up mechanism, the contribution of the projected RGB color prior is controlled by a learnable scalar parameter β∈R. The parameter β is initialized to zero and its value is learned during training. The final LCP-Conv output is then defined by Equation (18):

(18) $$Y_{\mathrm{out}}=Y+\alpha_{t}\beta \;\tilde{p}.$$

In Equation (18), Y represents the feature map generated by the standard convolutional path, $\tilde{p}$ represents the spatially expanded form of the projected RGB color prior, $\alpha_{t}$ is the linear warm-up coefficient defined in Equation (17), and β is the learnable scalar parameter controlling the contribution of the prior branch. In this additive structure, the standard convolutional feature representation is preserved, while the dataset-specific RGB color prior is incorporated through the additional term $\alpha_{t}\beta \tilde{p}$.

The RGB color prior used by LCP-Conv is computed once from the annotated lemon regions of the training set and remains fixed during inference. The same fixed prior is also used during validation. Therefore, the prior is not recalculated for each unseen input image, and no additional inference-time preprocessing is required to extract or update the color prior. When applying the model to a new dataset with substantially different imaging or color characteristics, the prior can be recalculated once from the corresponding training data to reflect the new dataset-level color distribution. This recalculation is not required for unseen images drawn from the same target distribution as the training data.

**Figure 7:**
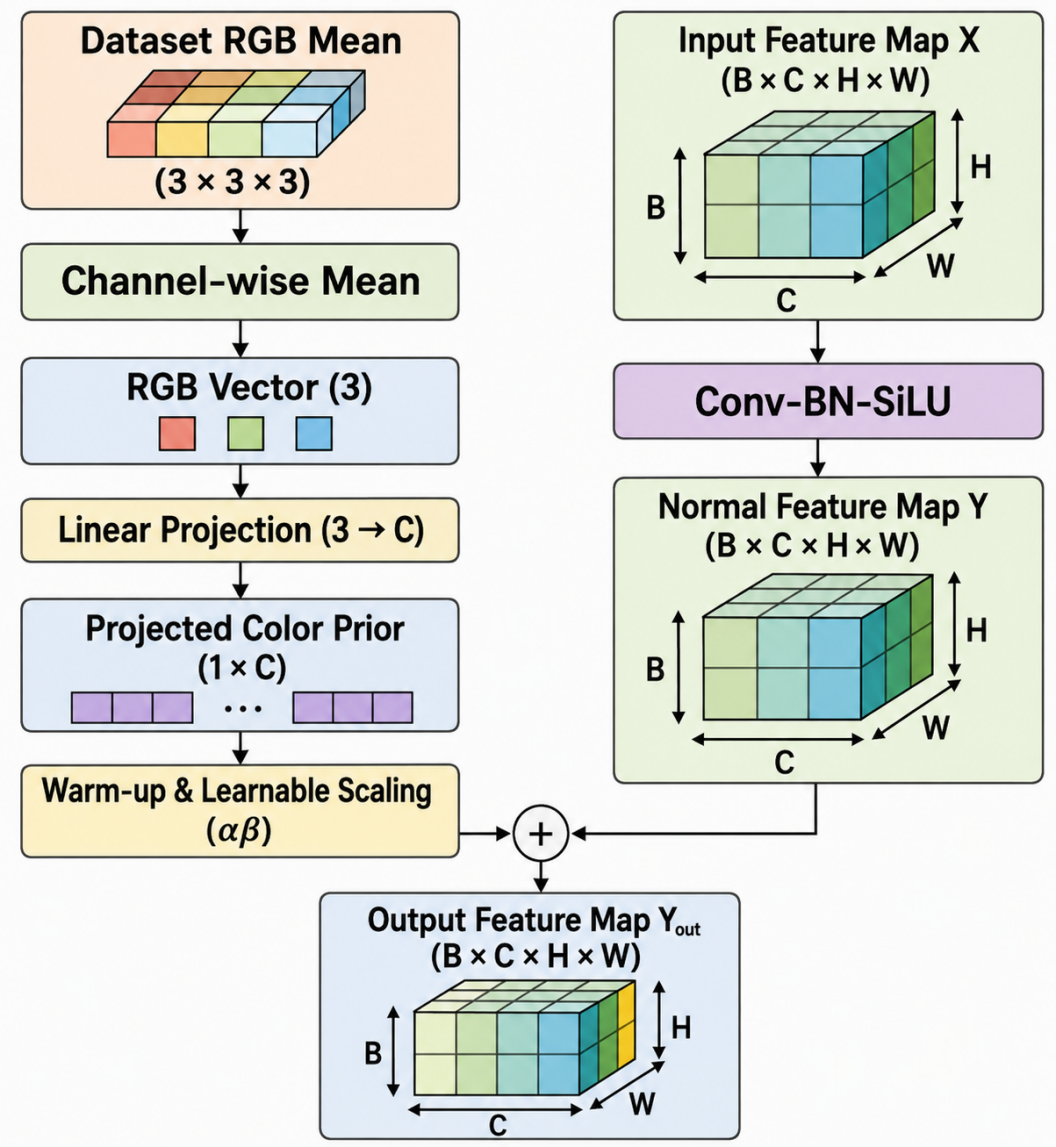
Structure of the proposed Lemon Color Prior Convolution (LCP-Conv) module. The standard Conv–BN–SiLU feature extraction path is combined with a parallel branch that processes the dataset-specific RGB color prior. The three-dimensional RGB prior is mapped to the output channel dimension through a learnable linear projection and incorporated into the convolutional feature map using the linear warm-up coefficient and the learnable scalar parameter.

The overall structure of the LCP-Conv module, defined by Equations (15)–(18), is shown in Figure 7. The module consists of the standard Conv–BN–SiLU feature extraction path and a parallel branch that processes the dataset-specific RGB color prior. The projected prior representation is incorporated into the convolutional feature map through the linear warm-up coefficient and the learnable scalar parameter.

### 4.2. LCP-Conv Integration and Small Object-Oriented Detection Structure

The position of the LCP-Conv module within LemonDet was determined by considering the object-scale distribution of the training data. As reported in Section 3.3, 69.55% of the lemon instances in the augmented training data satisfy the small-object criterion defined in Equation (9). This high proportion of small objects motivated the integration of the color prior at an early feature level with high spatial resolution. Accordingly, LCP-Conv was integrated into the P2 feature level of the LemonDet backbone. Since the P2 level has a higher spatial resolution than deeper feature levels, it provides a feature representation in which fine-grained spatial information associated with small objects can be retained before further downsampling. Thus, the dataset-specific RGB color prior $p_{RGB}$ defined in Equation (6) is incorporated into the early features at the P2 level through the LCP-Conv operations defined in Equations (16) and (18).

In addition to LCP-Conv integration, the multi-scale detection structure was reconfigured for small object detection. While object predictions in the standard YOLO11n architecture are generated from the P3, P4, and P5 feature levels [15], LemonDet was configured to generate predictions from the P2, P3, and P4 detection levels. Thus, the high-spatial-resolution P2 feature map is directly incorporated into the detection process, while the lower-spatial-resolution P5 detection level is excluded.

For an input image of size 640×640 pixels, LemonDet’s P2, P3, and P4 detection levels correspond to feature maps with spatial resolutions of 160×160, 80×80, and 40×40, respectively. The effective stride values for these levels are 4, 8, and 16, respectively. Specifically, the inclusion of the P2 level with a stride of 4 in the detection head enables high-resolution P2 features to contribute directly to object prediction.

**Figure 8:**
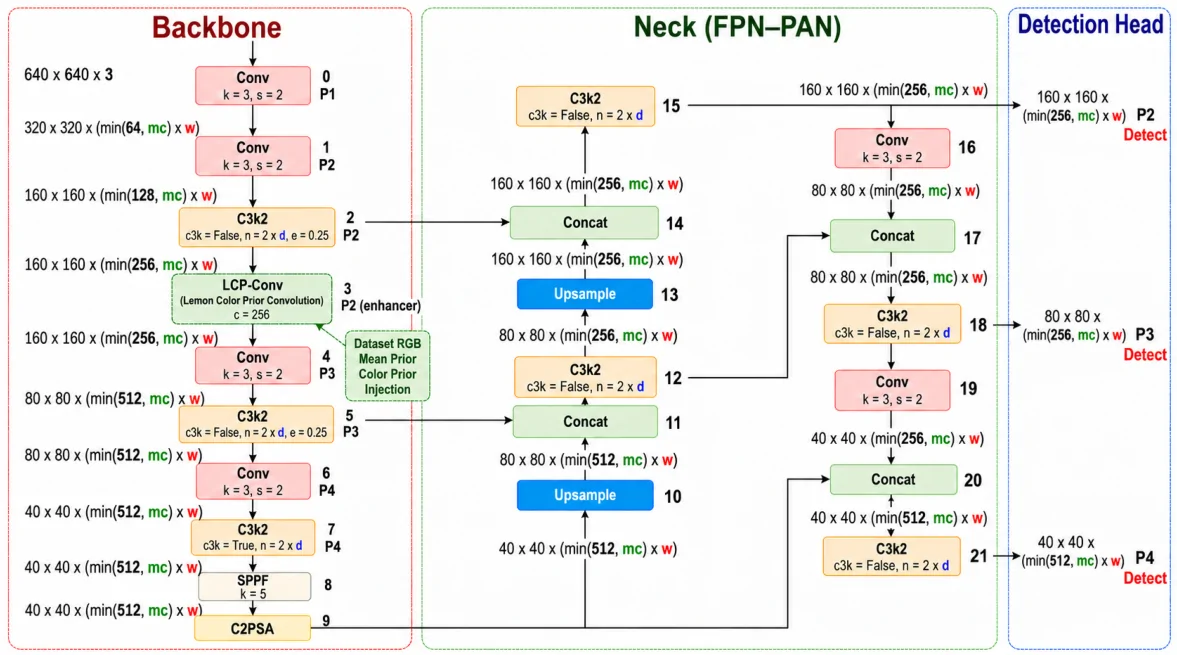
Proposed LemonDet architecture. The LCP-Conv module is integrated into the P2 level of the backbone to incorporate the dataset-specific RGB color prior into early feature extraction. The standard P3–P5 detection structure is reorganized as P2–P4, enabling high-resolution P2 features to contribute directly to the detection process.

Consequently, LemonDet incorporates two complementary architectural changes to the basic YOLO11n architecture: (i) the transfer of the dataset-specific RGB color prior to the early features at the P2 level via LCP-Conv, as defined in Equation (18), and (ii) the reorganization of the standard P3–P5 detection structure into P2–P4. The first modification incorporates dataset-specific color information into the feature extraction process, while the second enables high-resolution features to contribute directly to small object detection. The general structure of the proposed LemonDet architecture is shown in Figure 8.

## 5. Experimental Results

This section presents the experimental results of the proposed LemonDet model and examines the effects of the developed components on detection performance. Precision (P), Recall (R), F1-score, mAP50, and mAP50-95 metrics were used in the evaluation. First, the contributions of the components forming LemonDet were evaluated step-by-step, followed by an examination of the model’s convergence behavior during training. LemonDet was then compared with baseline YOLO models and representative non-YOLO detection architectures. Finally, the model’s performance was evaluated in detail through Precision-Recall and F1-score, confusion matrix, and sample detection results.

RGB-based color normalization using the dataset-specific RGB channel statistics obtained from the annotated lemon regions in the training data was first applied to the baseline YOLO11n model. In the next stage, the annotation-guided local enhancement approach for small objects described in Section 3.3 was incorporated. To separately evaluate the architectural contributions, the standard P3–P5 detection structure was then reconfigured as P2–P4 without LCP-Conv, while keeping the preprocessing pipeline and training settings unchanged. An additional P2–P4 configuration with a learnable bias term was also evaluated to examine whether the improvement provided by LCP-Conv could be attributed simply to the introduction of an additional learnable additive component. Finally, the complete LemonDet architecture, incorporating LCP-Conv at the P2 level, was evaluated under the same experimental conditions.

The ablation results in Table 3 allow the contribution of annotation-guided local enhancement to be examined separately from RGB-based color normalization. Under the same YOLO11n architecture, RGB-based color normalization achieved a Precision of 87.2%, Recall of 75.7%, F1-score of 81.0%, mAP50 of 86.0%, and mAP50-95 of 53.3%. When annotation-guided local enhancement was subsequently added while keeping the architecture unchanged, Recall increased by 1.8 percentage points, F1-score by 0.8 points, mAP50 by 1.0 point, and mAP50-95 by 0.2 points, while Precision decreased by 0.7 points. These results show that the local enhancement provides an additional contribution beyond RGB-based color normalization, particularly in terms of Recall and mAP50. When the standard P3–P5 detection structure was replaced with P2–P4 without LCP-Conv, Recall increased to 78.1%, F1-score to 82.0%, mAP50 to 87.9%, and mAP50-95 to 53.8%. These results indicate that incorporating the high-resolution P2 detection level provides a measurable, although moderate, improvement in the evaluated metrics.

The P2–P4 configuration with a learnable bias achieved 86.8% Precision, 78.7% Recall, 82.6% F1-score, 87.0% mAP50, and 53.9% mAP50-95. In comparison, the complete LemonDet configuration incorporating LCP-Conv at the P2 level achieved 87.2% Precision, 81.6% Recall, 84.3% F1-score, 89.2% mAP50, and 54.8% mAP50-95. Relative to the P2–P4 configuration without LCP-Conv, these results correspond to improvements of 3.5, 2.3, 1.3, and 1.0 percentage points in Recall, F1-score, mAP50, and mAP50-95, respectively. The higher performance of the complete LCP-Conv configuration compared with both the P2–P4-only and learnable-bias configurations indicates that the observed gain cannot be attributed solely to the detection-head restructuring or to the addition of a generic learnable term.

**Figure 9:**
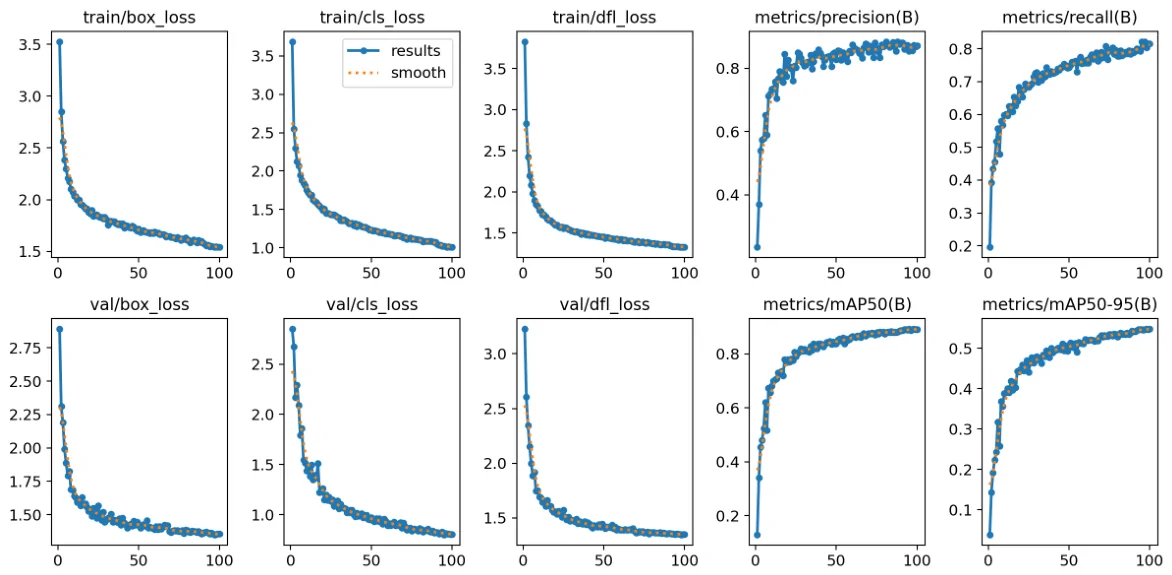
Training and validation results of the LemonDet model over 100 epochs. The top row shows the change in training losses and Precision and Recall values, and the bottom row shows the change in validation losses and mAP50 and mAP50-95 values according to epochs.

To examine LemonDet’s behavior during the training process, the changes in training and validation losses, as well as Precision, Recall, mAP50, and mAP50-95 values across epochs are shown in Figure 9. At the beginning of the training process, a rapid decrease was observed in box, classification, and distribution focal loss values, while this decrease continued more gradually in subsequent epochs. A similar trend was observed in validation losses. In contrast, Precision, Recall, mAP50, and mAP50-95 values increased rapidly, especially in the first epochs, and reached a more stable region in the later stages of training. The similar trends observed in the training and validation losses, together with the absence of a marked deterioration in the validation metrics toward the end of training, indicate a stable training process under the experimental conditions used.

Comparative results obtained to evaluate LemonDet’s performance against baseline YOLO models are presented in Table 4. Despite having fewer parameters than the compared models (0.99 million), LemonDet achieved 87.2% Precision, 81.6% Recall, 84.3% F1-score, 89.2% mAP50, and 54.8% mAP50-95. In particular, the higher Recall and mAP50 values indicate improved detection performance compared with the baseline YOLO models under the experimental conditions used.

To extend the comparative evaluation beyond YOLO-based models, LemonDet was also compared with RetinaNet and Faster R-CNN, both using an R50-FPN backbone, as well as EfficientDet-D0. These models were included as representative non-YOLO detection architectures. The results are presented in Table 5.

As shown in Table 5, LemonDet achieved higher mAP50 and mAP50-95 values than RetinaNet and Faster R-CNN under the evaluated experimental conditions. These results extend the comparative evaluation beyond the YOLO family and show that the performance advantage observed for LemonDet is also maintained against the evaluated non-YOLO detection architectures. All non-YOLO baseline models were implemented in PyTorch and trained in the Google Colab environment using an NVIDIA Tesla T4 GPU. RetinaNet (R50-FPN), Faster R-CNN (R50-FPN), and EfficientDet-D0 were initialized with pretrained weights and trained for 100 epochs using an input resolution of 640×640 pixels. AdamW was used as the optimizer for all three models with a batch size of 8. The initial learning rates were set to $1\times 10^{-4}$ for RetinaNet, $5\times 10^{-3}$ for Faster R-CNN, and $2\times 10^{-4}$ for EfficientDet-D0, while a weight decay of $5\times 10^{-4}$ was used for all models. RetinaNet and EfficientDet-D0 employed a CosineAnnealingLR scheduler, whereas Faster R-CNN employed a StepLR scheduler. For all three non-YOLO baselines, the checkpoint with the lowest validation loss was retained for evaluation.

LemonDet differs from the standard YOLO11n not only in detection performance but also in architectural complexity and computational requirements. Table 6 compares the two architectures in terms of detection levels, number of layers, parameter count, model size, inference time, FPS, GFLOPs, and detection performance. Inference-time measurements were performed on an NVIDIA Tesla T4 GPU using an input resolution of 640×640 pixels and a batch size of 1. FP32 numerical precision was used during the timing experiments, and warm-up runs were performed before recording the measurements. The reported latency includes preprocessing, model inference, and post-processing. LemonDet contains 0.994 million parameters and has a model size of 2.4 MB, compared with 2.58 million parameters and 5.5 MB for YOLO11n. The use of the high-resolution P2 detection level increases the computational cost from 6.4 to 13.5 GFLOPs and the inference time from 13.0 to 20.8 ms/image, corresponding to 76.9 and 48.1 FPS, respectively. Despite this additional computational cost, LemonDet improves mAP50 and mAP50-95 while maintaining real-time inference under the evaluated conditions.

**Figure 10:**
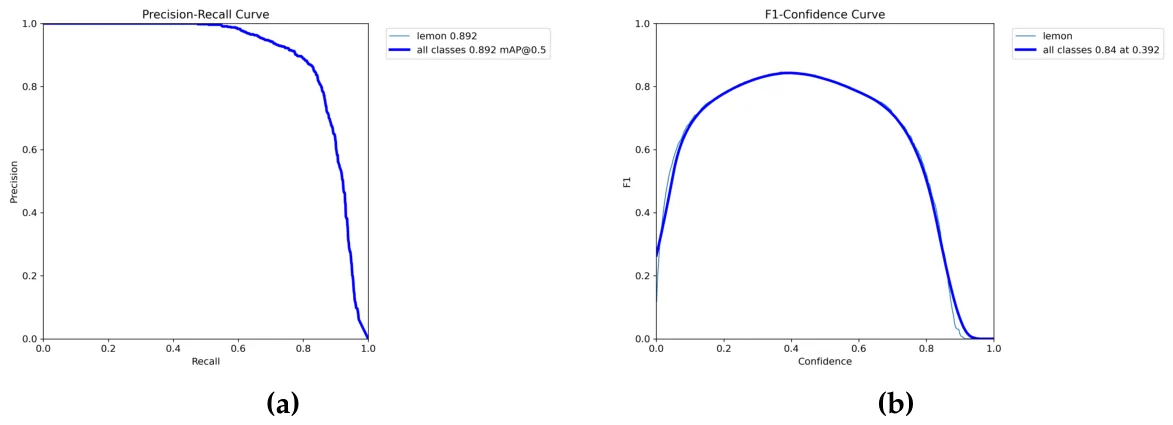
LemonDet performance curves on validation data: (**a**) Precision–Recall curve and (**b**) F1-score.

Single-point performance is given in the tables. In addition to the values, LemonDet’s behavior at different decision thresholds was examined through Precision-Recall and F1-score. The Precision-Recall curve in Figure 10a shows that LemonDet reached an AP value of 0.892 under the IoU = 0.50 condition. The high level of preservation of the Precision value over a wide Recall range indicates that the model can maintain a significant portion of correct detections as Recall increases. A more pronounced decrease in the Precision value occurs in the region where the Recall value approaches its upper limit.

The F1-score in Figure 10b shows how the balance between Precision and Recall changes depending on the confidence threshold. The maximum F1-score value was obtained at approximately 0.84, and this occurred at a confidence threshold of approximately 0.392. This point represents the confidence region where the highest F1-score balance between Precision and Recall is achieved on the available validation data.

**Figure 11:**
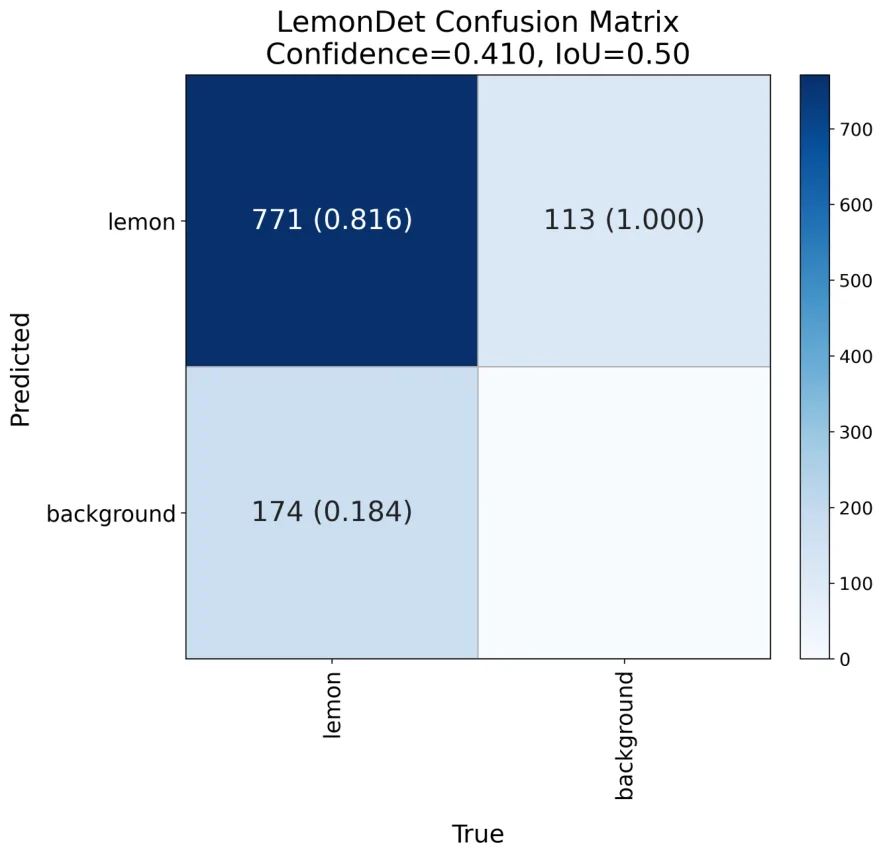
Confusion matrix of LemonDet on the validation set comprising 61 images and 945 annotated instances. Raw counts are shown together with the corresponding normalized values in parentheses. Normalization was performed column-wise with respect to each true class. The matrix was generated using a confidence threshold of 0.410 and an IoU matching threshold of 0.50.

To evaluate LemonDet’s correct detections as well as false-positive and missed detections, the confusion matrix is presented in Figure 11. At a confidence threshold of 0.410 and an IoU matching threshold of 0.50, LemonDet produced 771 true-positive (TP), 113 false-positive (FP), and 174 false-negative (FN) detections on the validation set. These values correspond to a Precision of 87.2%, Recall of 81.6%, and F1-score of 84.3%, consistent with the results reported in Table 4. The remaining false-positive and missed detections indicate that challenging conditions such as complex backgrounds, dense object clustering, overlap, and visual similarities between lemons and surrounding regions can still affect detection performance.

To further examine LemonDet’s detection performance with respect to object scale, a size-stratified analysis was conducted on the validation set, and the results are presented in Table 7. Based on bounding-box area, objects were categorized as small (≤$32^{2}$ pixels), medium (>$32^{2}$ and ≤$96^{2}$ pixels), and large (>$96^{2}$ pixels). Among the 945 annotated lemon instances, 338 (35.77%) were classified as small, 580 (61.38%) as medium, and 27 (2.86%) as large. LemonDet correctly detected 222 small, 523 medium, and 26 large objects, corresponding to Recall values of 65.68%, 90.17%, and 96.30%, respectively. The results show that Recall decreases as object size decreases, confirming that small lemons constitute the most challenging scale category in the validation set.

**Figure 12:**
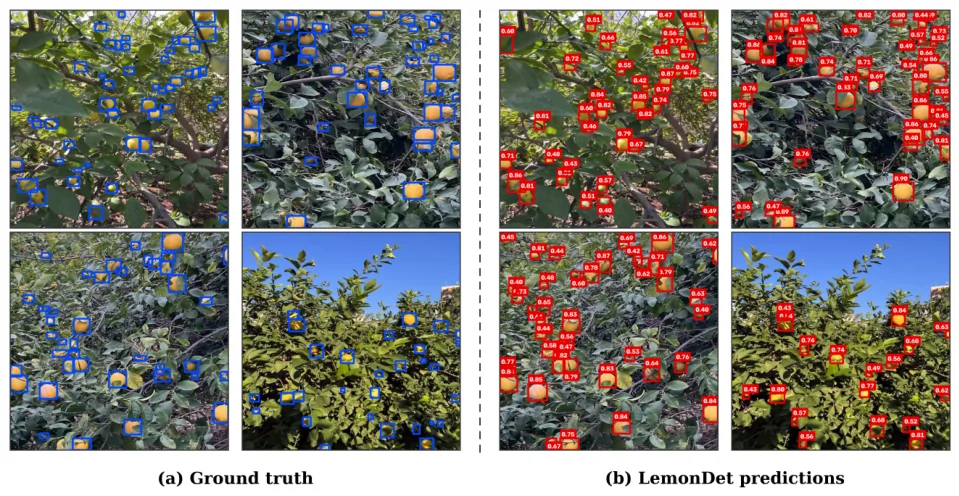
Representative detection results on the LemonDet validation set: (**a**) ground-truth bounding box annotations and (**b**) predictions and confidence values obtained by LemonDet.

In addition to the quantitative evaluation, sample ground-truth annotations and model predictions from the validation set were compared in Figure 12 to visually examine LemonDet’s detection behavior under different image conditions. The visual results show that the model can localize lemons of different sizes and a significant portion of objects that are densely located within the same image. In particular, the ability to detect many lemon objects that partially overlap with leaves and occupy a relatively small area within the image is consistent with the Recall and mAP50 values obtained in the quantitative results. However, in some areas where there is dense overlap or the visible area of the object is quite limited, predictions with low confidence values and missed objects are observed.

When the quantitative and qualitative results obtained are evaluated together, LemonDet’s underlying YOLO11n structure shows higher performance, particularly in the Recall, F1-score, and mAP50 metrics. The extended ablation results show that the P2–P4 detection restructuring provides a moderate improvement over the preprocessing-only configuration, while the subsequent integration of LCP-Conv produces a larger additional gain, particularly in Recall, F1-score, and mAP50. The learnable-bias control further indicates that this improvement cannot be attributed solely to the introduction of an additional learnable additive term. However, mismatched predictions observed in the confusion matrix and qualitative results reveal areas for model improvement, particularly in cases of dense overlap and complex background conditions.

## 6. Discussion

Experimental results show that LemonDet provides higher detection performance than basic YOLO models for the lemon detection problem under consideration. This improvement should be interpreted in conjunction with the object scale distribution of the dataset, as 69.55% of the training samples meet the adopted small-object criterion. Therefore, high-resolution P2 features, dataset-specific color information, and annotation-guided local enhancement were specifically designed for this data distribution. The extended ablation results provide a clearer distinction between the architectural contributions. Reconfiguring the detection structure from P3–P5 to P2–P4 provided a moderate improvement, while the subsequent integration of LCP-Conv at the P2 level resulted in a larger additional gain, particularly in Recall, F1-score, and mAP50. The learnable-bias control did not reproduce the performance of the complete LCP-Conv configuration, indicating that the observed improvement cannot be explained solely by the addition of a generic learnable additive term.

Dataset-specific RGB color prior represents another problem-focused component of LemonDet. The results show that color statistics derived from labeled lemon regions can provide complementary information for detection. However, these statistics are specific to the training data and should not be assumed to remain valid under different fruit types, ripening stages, cameras, or lighting conditions. The current experiments do not explicitly quantify the sensitivity of LemonDet to controlled shifts in the underlying color distribution. For a new dataset, the RGB color prior should therefore be recalculated from the corresponding training data. In addition, the soft color normalization strategy and the learnable scaling mechanism in LCP-Conv control the influence of the color prior rather than directly forcing the feature representation to follow fixed RGB statistics.

The confusion matrix, size-stratified analysis, and qualitative results also reveal the remaining limitations of the model. Recall decreased with object size, with small objects showing lower values than medium and large objects, confirming that small lemons remain the most challenging scale category. False-positive and missed detections also remain present, particularly under challenging visual conditions. Heavy overlap, limited visible object areas, and complex vegetation were among the more challenging conditions observed in the qualitative samples. These results indicate that improving the detection of very small and partially visible objects and reducing background-induced false positives remain important directions for further improvement.

Despite these limitations, LemonDet achieved higher mAP50 and mAP50-95 values while maintaining a compact architecture with approximately 0.99 million parameters. Accordingly, the lightweight characterization of LemonDet refers specifically to its reduced parameter count and model storage size, rather than to a reduction in FLOPs or inference latency. Although LemonDet is parameter- and storage-efficient relative to YOLO11n, the incorporation of the high-resolution P2 detection level increases its computational cost and inference latency. Nevertheless, LemonDet achieved an inference speed of 48.1 FPS under the evaluated hardware and experimental conditions, indicating that real-time processing remains feasible. However, the experiments were performed on a single lemon dataset and a single object class, and the present study does not include an independent test set, repeated-seed evaluation, or external validation on an independently collected dataset. Therefore, the reported results should be interpreted as validation-set performance under the current experimental setting. Further evaluation is needed on different orchards, camera systems, seasons, lighting conditions, lemon varieties, acquisition altitudes, object densities, occlusion levels, and camera-object distances to assess the generalizability and robustness of the method under systematically controlled conditions. Future studies will address these limitations through independent test data, repeated experiments with different random seeds, and external validation across different orchards and acquisition conditions. Extending LCP-Conv to multi-class detection also requires further research on how to represent and integrate class-specific color priors.

## 7. Conclusions

In this study, a YOLO11n-based LemonDet model is proposed to improve the detection performance of small lemon objects in particular. Considering the high proportion of small objects in the training data, the standard P3–P5 detection structure was restructured as P2–P4. Furthermore, dataset-specific RGB color prior obtained from labeled lemon regions was incorporated into early feature extraction via LCP-Conv, and annotation-guided local enhancement was applied to small object regions.

The experimental results show that, LemonDet achieved 87.2% Precision, 81.6% Recall, 84.3% F1-score, 89.2% mAP50, and 54.8% mAP50-95. Ablation results showed that improving detection performance was achieved by gradually adding RGB-based color normalization and annotation-guided local enhancement components, and that the LemonDet configuration including all components achieved the highest overall detection performance. Furthermore, LemonDet achieved higher detection performance than the benchmark YOLO models compared, while maintaining a compact architecture with 0.99 million parameters.

The results suggest that considering dataset-specific color information and object scale distribution in model design can be beneficial for small object-weighted lemon detection. However, the generalizability of the method under different orchards, camera systems, lighting conditions, and lemon varieties needs to be evaluated. Future studies will evaluate LemonDet on different agricultural datasets, examine different forms of color prior information, and develop improvements to reduce false positives and missed detections, particularly under conditions of dense overlap.

## Figures and Tables

**Table 1:** Distribution of images and labeled lemon objects in the dataset according to training and validation sets.

Data	Total	Training	Validation
Image	436	375	61
Lemon object	9469	8524 (%90)	945 (%10)

**Table 2:** Dataset-specific RGB color prior obtained from the training data.

Channel	Mean Value
Red (R)	0.434
Green (G)	0.433
Blue (B)	0.239

**Table 3:** Ablation study of the preprocessing and architectural components of the proposed LemonDet approach.

Model	Architecture	Preprocessing	P (%)	R (%)	F1-Score (%)	mAP50 (%)	mAP50-95 (%)
YOLO11n	P3–P5	None	85.4	74.1	79.3	84.3	52.3
YOLO11n	P3–P5	CN	87.2	75.7	81.0	86.0	53.3
YOLO11n	P3–P5	CN + LE	86.5	77.5	81.8	87.0	53.5
YOLO11n	P2–P4	CN + LE	86.2	78.1	82.0	87.9	53.8
YOLO11n	P2–P4 + LB	CN + LE	86.8	78.7	82.6	87.0	53.9
**LemonDet**	**P2–P4 + LCP-Conv**	**CN + LE**	**87.2**	**81.6**	**84.3**	**89.2**	**54.8**

CN: RGB-based Color Normalization; LE: Annotation-Guided Local Enhancement; LB: Learnable Bias.

**Table 4:** Performance comparison of LemonDet with baseline YOLO models.

Model	Params	P (%)	R (%)	F1-Score (%)	mAP50 (%)	mAP50-95 (%)
YOLOv8n	3.01M	85.5	77.0	81.0	86.0	53.3
YOLO11n	2.58M	86.5	77.5	81.8	87.0	53.5
**LemonDet**	**0.99M**	**87.2**	**81.6**	**84.3**	**89.2**	**54.8**

**Table 5:** Performance comparison of LemonDet with representative YOLO and non-YOLO detection architectures.

Model	Precision (%)	Recall (%)	F1-Score (%)	mAP50 (%)	mAP50-95 (%)
RetinaNet (R50-FPN)	73.4	84.4	78.5	85.5	50.4
Faster R-CNN (R50-FPN)	84.4	80.3	82.3	83.3	50.3
EfficientDet-D0	87.4	76.2	81.4	84.2	50.3
YOLO11n	86.5	77.5	81.8	87.0	53.5
**LemonDet**	**87.2**	**81.6**	**84.3**	**89.2**	**54.8**

**Table 6:** Detection performance, model complexity, and computational characteristics of YOLO11n and LemonDet.

Model	Detection Levels	Layers	Params	Model Size	Inference Time (ms/Image)	FPS	GFLOPs	mAP50/mAP50-95 (%)
YOLO11n	P3–P5	100	2.58M	5.5 MB	13.0	76.9	6.4	87.0/53.5
**LemonDet**	**P2–P4**	**89**	**0.994M**	**2.4 MB**	**20.8**	**48.1**	**13.5**	**89.2/54.8**

**Table 7:** Size-stratified detection performance of LemonDet on the validation set.

Object Size	GT Objects	TP	FN	Recall (%)
Small	338	222	116	65.68
Medium	580	523	57	90.17
Large	27	26	1	96.30
**Overall**	**945**	**771**	**174**	**81.59**

Object-size categories were defined according to bounding-box area as small (≤$32^{2}$ pixels), medium (>$32^{2}$ and ≤$96^{2}$ pixels), and large (>$96^{2}$ pixels). The analysis was performed at a confidence threshold of 0.410 and an IoU matching threshold of 0.50, consistent with the confusion-matrix evaluation.

## Data Availability

The dataset used in this study was originally collected and annotated by the authors. The data are available from the corresponding author upon reasonable request.

## Abbreviations

The following abbreviations are used in this manuscript:

LCP-Conv	Lemon Color Prior Convolution
RGB	Red, Green, Blue
CLAHE	Contrast Limited Adaptive Histogram Equalization
CIELAB	Commission Internationale de l’Éclairage L*a*b* color space
YOLO	You Only Look Once
mAP	Mean Average Precision
IoU	Intersection over Union
P	Precision
R	Recall
F1-score	F1-score
